# Occipitocervical Fusion *via* Cervical Pedicle Fixation Assisted with O‐arm Navigation

**DOI:** 10.1111/os.12704

**Published:** 2020-07-22

**Authors:** Yu‐cheng Wang, Zhang‐zhe Zhou, Bin Wang, Kai Zhang, Hao Chen, Kang‐wu Chen, Hai‐qing Mao

**Affiliations:** ^1^ Department of Orthopaedic Surgery The First Affiliated Hospital of Soochow University Suzhou China; ^2^ Department of Orthopaedic Surgery Suzhou Hospital of Traditional Chinese Medicine Suzhou China; ^3^ Department of Orthopaedic Surgery The Second Affiliated Hospital of Xuzhou Medical University Xuzhou China

**Keywords:** Cervical pedicle screw fixation, O‐arm navigation, Occipitocervical fusion

## Abstract

**Objective:**

To describe the clinical outcomes of occipitocervical fusion (OCF) using cervical pedicle fixation with assistance of O‐arm navigation and present its clinical feasibility.

**Methods:**

From January 2015 to December 2016, eight patients with a variety of diagnoses were surgically treated with occipitocervical fusion using cervical pedicle screws under O‐arm navigation. All patients received full workup consisting of clinical and radiological assessments. Perioperative parameters including operating time, intraoperative blood loss, postoperative complications, surgical outcomes were recorded. Postoperative data were acquired resorting to the scheduled follow‐up 3, 6 and 12 months after their discharge and annually afterwards. The Japanese Orthopaedic Association (JOA) Scores and American Spinal Injury Association (ASIA) Scale were used to evaluate neurological function. The accuracy of screw placement was classified according to a modified classification of Gertzbein and Robbins. The fusion status was evaluated in reference to the Bridwell's posterior fusion grades.

**Results:**

The patient cohort comprised of five males and three females, with the average age of 51.9 years (range from 18 to 74 years). The patients all showed indications for OCF and were performed with polyaxial screws through cervical pedicles. The average operation time was 274 min (range from 226 to 380 min), with the intraoperative blood loss of 437.5 mL and the blood transfusion volume of 481.3 mL. The average follow‐up time was 23.5 months (range from 17 to 32 months). All patients exhibited radiographic evidence of osseous fusion by X‐ray and computed tomography (CT) at the final follow‐up. No neurovascular complications were found during the follow‐up time, and the clinical symptoms were observed to be significantly improved in all the patients. Thirty‐four cervical pedicle screws were implanted within the eight patients, with the accuracy of cervical pedicle screw placements as 94.1% (32/34), among which, two pedicle screws were found to broken through the cervical pedicles that were evaluated as Grade II.

**Conclusions:**

Occipitocervical fusion via cervical pedicle fixation assisted with O‐arm navigation is a feasible and safe procedure with a vast range of indications.

## Introduction

Occipitocervical fusion (OCF) is used to treat craniocervical pathologies losing biomechanical stability. These disorders generate clinical symptoms, including severe occipital neck pain, restricted neck movement, myelopathy, which significantly reduce patients' quality of life. Traumatic dislocation or subluxation, congenital malformation, craniovertebral junction (CVJ) reconstruction after decompression are the most common indications of occipitocervical fusion^1^. To achieve rigid immobilization, rod and wire constructs have been largely substituted by screw and rod designs recently^2^. The screws could be implanted via multiple techniques including inserting through cervical pedicles/pars/lateral masses or employing transarticular and intralaminar screws. The cervical pedicle screws were believed to provide maximum strength for mechanical stability, which is of great importance for the internal fixation^3^, ^4^.

However, traditional surgical technique guided with C‐arm fluoroscopy is challenging and vulnerable to damage vertebral artery, spinal cord, or nerve root. These iatrogenic injuries might lead to intraoperative massive hemorrhage, cardiopulmonary arrest, high paraplegia, or paralysis. Recently, several studies have reported that O‐arm navigation is a safe, feasible, and efficient application in lumbar surgeries^5^, ^6^, while the experience of usage in upper cervical spine and CVJ remains insufficient. In this study, we present eight patients treated with O‐arm navigation assisted OCF via cervical pedicle fixation.

In this study, the results of treatment were evaluated by retrospective follow‐up of patients: (i) to evaluate the accuracy of screw placement under O‐arm navigation; (ii) to observe the fusion status after operation; and (iii) to assess the clinical outcome of occipitocervical fusion using cervical pedicle fixation under O‐arm navigation.

## Materials and Methods

### *Patient Population*

This retrospective study reviewed eight patients performing occipitocervical fusion in our institution between January 2015 and December 2016. All patients were operated with occipitocervical fusion using cervical pedicle screws under O‐arm navigation by the same surgical team. All patients received full workup consisting of clinical and radiological assessments. Perioperative parameters including operating time, intraoperative blood loss, postoperative complications, surgical outcomes were recorded. Postoperative data were acquired resorting to the scheduled follow‐up 3, 6, and 12 months after their discharge and annually afterwards. Inclusion criteria were as follows: (i) patients with instability of craniocervical lesions; (ii) occipitocervical fusion via cervical pedicle fixation assisted with O‐arm navigation; (iii) comparison of preoperative and postoperative status; (iv) evaluation of surgical procedure and postoperative status; and (v) retrospective study. Exclusion criteria were as follows: (i) lost to follow‐up; (ii) infections, psychiatric disorders, coagulation disorders; and (iii) cancer patients.

### *The Japanese Orthopaedic Association (JOA) Scores*

The highest score of JOA is 29, and the lowest is 0. Improvement rate = [(post‐treatment score − pre‐treatment score)/29 − pre‐treatment score] × 100%. The clinical treatment effect can be understood by the improvement rate. When the improvement rate is 100%, it is a cure. When the improvement rate is more than 60%, it is significantly effective. When the improvement rate is 25%–60%, it is effective. When the improvement rate is less than 25%, it is ineffective.

### *American Spinal Injury Association (ASIA) Scale*

American Spinal Injury Association (ASIA) Scale was used to evaluate neurological function. A = Complete: No motor or sensory function is preserved in the sacral segments S4‐S5. B = Incomplete: Sensory but not motor function is preserved below the neurological level and includes the sacral segments S4‐S5. C = Incomplete: Motor function is preserved below the neurological level, and more than half of key muscles below the neurological level have a muscle grade less than 3. D = Incomplete: Motor function is preserved below the neurological level, and at least half of key muscles below the neurological level have a muscle grade of 3 or more. E = Normal: motor and sensory functions are normal.

### *The Accuracy of Screw Placement*

The accuracy of screw placement was classified according to a modified classification of Gertzbein and Robbins^7^. Grade A is ideal screw position, grade B represents pedicle wall perforations <2 mm, grade C represents 2 ≤ pedicle wall perforations <4 mm, and grade D represents 4 ≤ pedicle wall perforations <6 mm.

### *The Fusion Status*

The fusion status was evaluated referring to the Bridwell's posterior fusion grades^8^. Grade I: bilateral solid trabecular transverse process and facet joint fusion; grade II: thick fusion block on one side, and difficult to see on the other side; grade III: suspicious defect in fusion mass; grade IV: bone graft clear absorption with fatigue of instrumentation. The fusion is defined as grade I or II.

**Figure 1:**
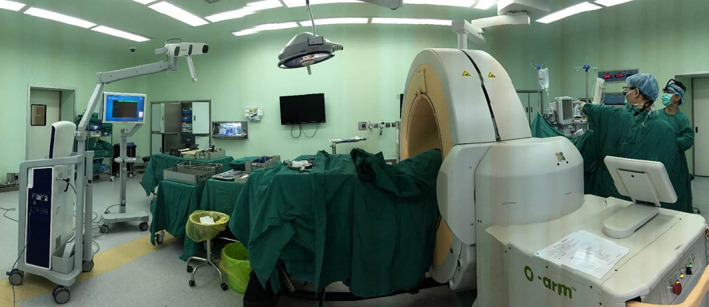
The surgical site and surgical equipment (O‐arm machine) were shown.

Our study protocol was approved by the Ethics Review Committee of the Institutional Review Board of the hospital. All the patients or families are informed of standard written consent to acquire their clinical information. This case series study has been reported in line with the PROCESS criteria^9^. The surgical site and surgical equipment (O‐arm machine) were shown in Fig. 1.

### *Surgical Procedure*

#### *Anesthesia and Position*

Under general anesthesia, the patients were placed in the prone position with their head fixed in slight flexion with head holder.

#### *Exposure*

A conventional midline incision was made from external occipital protuberance (EOP) to spinous process of the third cervical vertebrae. The posterior components were exposed using the classic subperiosteal technique with great caution to avoid injuring the greater occipital nerve. The facet joints of the C_2_ and C_3_ vertebrae were exposed for placement of the pedicle screws by blunt dissection.

#### *Placement and Fixation*

The posterior arch of the C1 vertebrae was exposed for adequate decompression or as a salvage backup to provide pedicles for fixation when indicated. Under O‐arm navigation, the entry point and orientation of the screws were adjusted to conform to the complex anatomy of cervical pedicle. Four screws were inserted strictly in place through the pedicles of C_2_ and C_3_ and another four screws were placed on both sides of EOP, below the superior nuchal line.

#### *Reconstruction*

After attaching two pre‐bent connecting rods to screws bilaterally, a final scan was conducted to evaluate the position of implantations, intraoperative reduction and/or decompression. A high‐speed grinder was used to polish posterior cortex of the occipital bone and cervical lamina whereupon bone grafting was performed.

According to the individual condition of each patient, prolonged segment fusion, deformity correction, C1 posterior arch excision, or laminoplasty was performed.

## Results

### *Patient Population*

The demographic features and clinical information for these patients are summarized in Table 1. The patient cohort comprised five males and three females, with an average age of 51.9 years (range, 18–74 years). The diversity of the patients’ diagnoses included most of the common indications of OCF (Table 1). All the patients received OCF via cervical pedicles using polyaxial screws without any salvage alternatives via other osseous channels. The average operation time was 274 min (range, 226–380 min, including two hybrid surgeries). The average intraoperative blood loss and transfusion volume were 437.5 mL and 481.3 mL, respectively. The average follow‐up time was 23.5 months (range, 17–32 months) (Table 2).

**TABLE 1:** Summary of Clinical Data for Patients performed OCF

Case	Age/Sex	Diagnosis	Etiology	Symptoms and sign	Duration of symptoms
1	74/M	CCS, CSM	Degenerative	Limb numbness; paresthesia; walking unstable	6 years
2	57/F	CCS, CSM	Degenerative	Upper limb numbness; walking unstable; severe limb weakness	20 year
3	63/M	Odontoid Nonunion	Traumatic	Restricted neck movement; limb weakness	50 years
4	18/F	BI	Congenital	Short neck, walking unstable	11 months
5	57/F	Deformity	Acquired	Neck pain; Limb numbness	6 months
6	36/M	OC dislocation	Traumatic	Neck pain; incomplete paralysis	4 h
7	42/M	OCD	Traumatic	Neck pain	7 days
8	68/M	OCI	Traumatic	Neck pain	3 days

BI, basilar invagination; CCS indicates cervical canal stenosis; CSM, cervical spondylotic myelopathy; OCD, occipitocervical dislocation; OCI, occipitocervical instability.

**TABLE 2:** Information about surgery

Case	Instrumentation	Operation time (min)	Blood loss /Transfusion (mL)	Follow‐up (months)
1	Oc, C_2_, C_3_; posterior decompression of C_1_; laminoplasty of C_4_‐C_7_	344	500/800	32
2	Oc, C_1_, C_3_, C_4_; laminoplasty of C_5_‐C_7_	380	600/1000	26
3	Oc, C_1_, C_2_	235	400/150	17
4	Oc, C_2_, C_3_, C_4_	273	800/1550	30
5	Oc, C_2_, C_3_; posterior decompression of C_1_;	250	100/0	20
6	Oc, C_2_, C_3_	240	450/200	23
7	Oc, C_2_, C_3_	245	400/150	22
8	Oc, C_2_, C_3_	226	250/0	18

### *The Japanese Orthopaedic Association (JOA) Scores*

The symptoms of the patients were improved and the surgical treatment was effective (Table 3). The improvement rate was 100% in two patients and 30% in the lowest one.

**TABLE 3:** JOA scores improvement rate (%) of the patients postoperatively and at the final follow‐up

Case	JOA	
	Post	Follow‐up
1	37.5	37.5
2	50	50
3	30	30
4	60	60
5	100	100
6	33.3	33.3
7	—	—
8	100	100

### *American Spinal Injury Association (ASIA) Scale*

No neurovascular complications occurred, and the clinical symptoms had improved for all patients (Table 4). Three patients recovered to grade E, four to grade D and one to grade C.

**TABLE 4:** ASIA classification of the patients with neurological deficits preoperatively and at the final follow‐up

	Postoperation					
Preoperation	A	B	C	D	E	Total
A	0	0	0	0	0	0
B	0	0	1	0	0	1
C	0	0	0	1	0	1
D	0	0	0	3	2	5
E	0	0	0	0	1	1
Total	0	0	1	4	3	8

### *The Accuracy of Screw Placement*

In all, thirty‐four cervical pedicle screws were implanted. The distribution and the accuracy grades of the screws were list in Table 5. The accuracy of cervical pedicle screw placements was 94.1%(32/34). Two pedicle screws were observed penetration less than 2mm and considered as Grade B.

**TABLE 5:** The accuracy of pedicle screws placement

Distribution	Accuracy grades			Total
	I	II	III	
C_1_	4	0	0	4
C_2_	13	1	0	14
C_3_	13	1	0	14
C_4_	2	0	0	2

### *The Fusion Status*

All patients exhibited radiographic evidence of osseous fusion by X‐ray and CT reconstruction at the final follow‐up. X‐ray and CT showed that the sequence of vertebral body was good. No pseudarthrosis and displacement in the vertebral body of all patients and no loosening or fracture of screws were found.

### *Illustrative Case*

**Figure 2:**
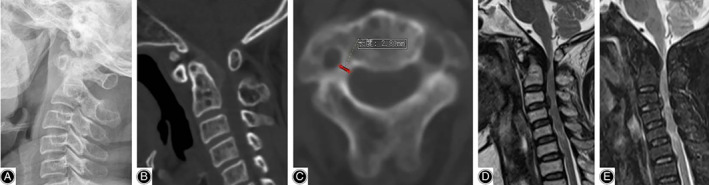
Preoperative images of the illustrative case. (A) Lateral radiograph showed slight deformity of the odontoid process. (B) CT on sagittal plane showed that the thickening of ligaments resulted in spinal stenosis. (C) CT on transverse plane showed excessive narrowness of the pedicles of C_2_. (D, E) MRI demonstrated severe spine canal stenosis and compression of the spinal cord.

A 57‐year‐old female patient had a sole complaint of upper limb numbness for almost 20 years. The symptom progressively aggravated and presented with manifestations of cervical myelopathy 1 month before she underwent the surgery. Physical examination revealed severe weakness and paresthesia of four limbs (of grade III), accompanied by tendon hyperreflexia. During the course of disease, no history of traumatic injury was acknowledged. Preoperative X‐ray indicated a mild deformity in the atlantoaxial joint, the interval between C_1_ posterior arch and C_2_ spinal process was slightly expanded and the odontoid process presented as a dorsal arch shape. Dynamic plain radiographs showed no signs of instability or dislocation/subluxation. The CT scan further confirmed the radiological changes above. Notably, we defined the pedicles of C_2_ as unsuitable working cannels according to scans on transversal plane (Fig. 2). Magnetic resonance imaging (MRI) illustrated an obvious compression on the ventral spinal cord. High‐intensity zone was clearly visible on sagittal plane of T2 and STIR sequence.

**Figure 3:**
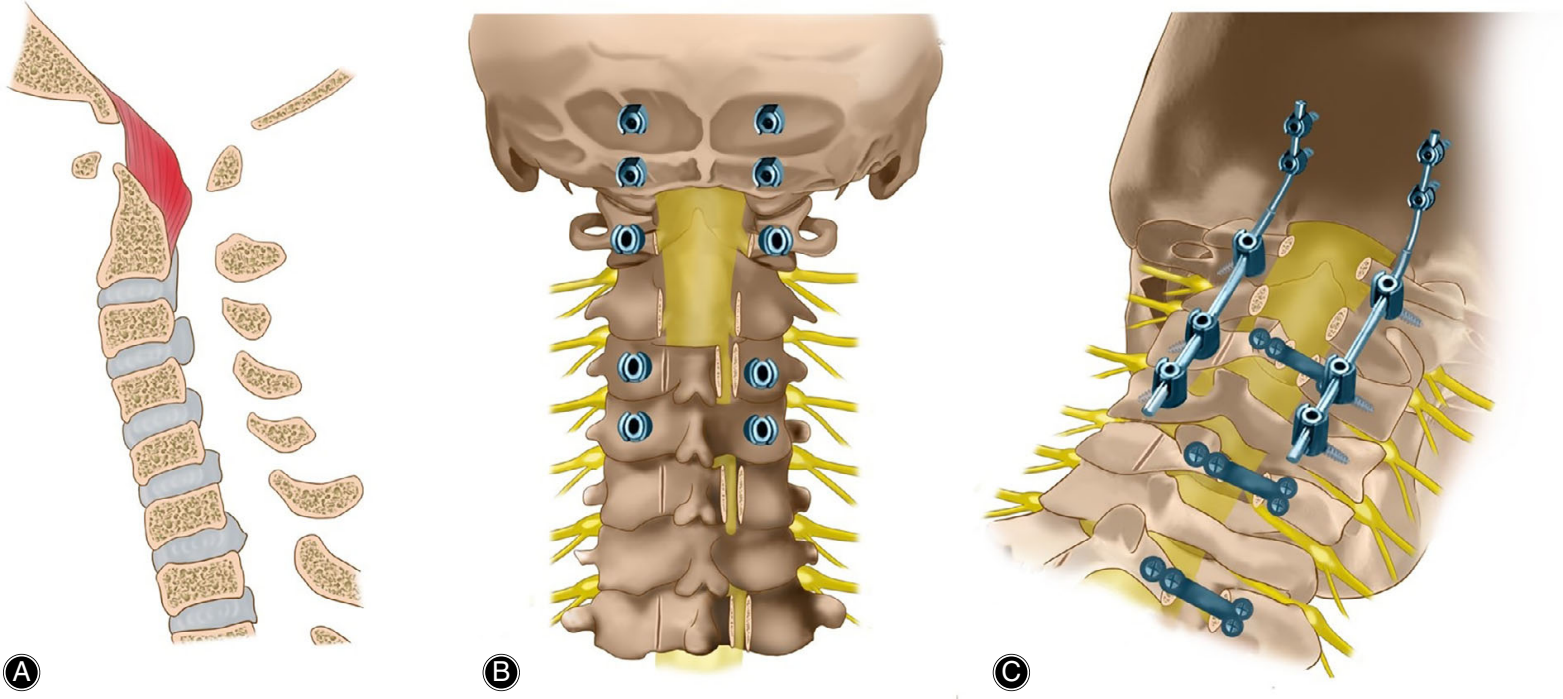
Diagrams description of New surgical technique: (A) The spinal cord of many segments is obviously compressed, especially C_1_‐C_2_; (B) The hybrid surgery‐OCF integrated with laminoplasty was performed from EOP to C_7_. The fixation through pedicles of C_1_, C_2_ and C_4_ was maneuvered and the occipital bone was partly bite off for sufficient decompression. Laminoplasty from C_3_ to C_7_ was operated and three micro plates were attached to the open door of C_3_, C_5_ and C_7_. (C) After attaching two pre‐bend connecting rods to screws bilaterally, a final scan was operated to evaluate the position of implantations and intraoperative reduction.

An extremely long‐segment incision from EOP to C_7_ was made to perform the hybrid surgery‐OCF integrated with laminoplasty. We did the fixation from EOP to C_4_, while the C_2_ pedicles were rechecked intraoperatively to be bypassed, instead we maneuvered the fixation through pedicles of C_1_. The occipital bone was partly bit off with the rongeur till the medulla is exposed for sufficient decompression, accompanied by laminoplasty from C_3_ to C_7_. Three micro plates were attached to the open door of C_3_, C_5_, and C_7_ to prevent from recurrence of stenosis (Fig. 3).

**Figure 4:**
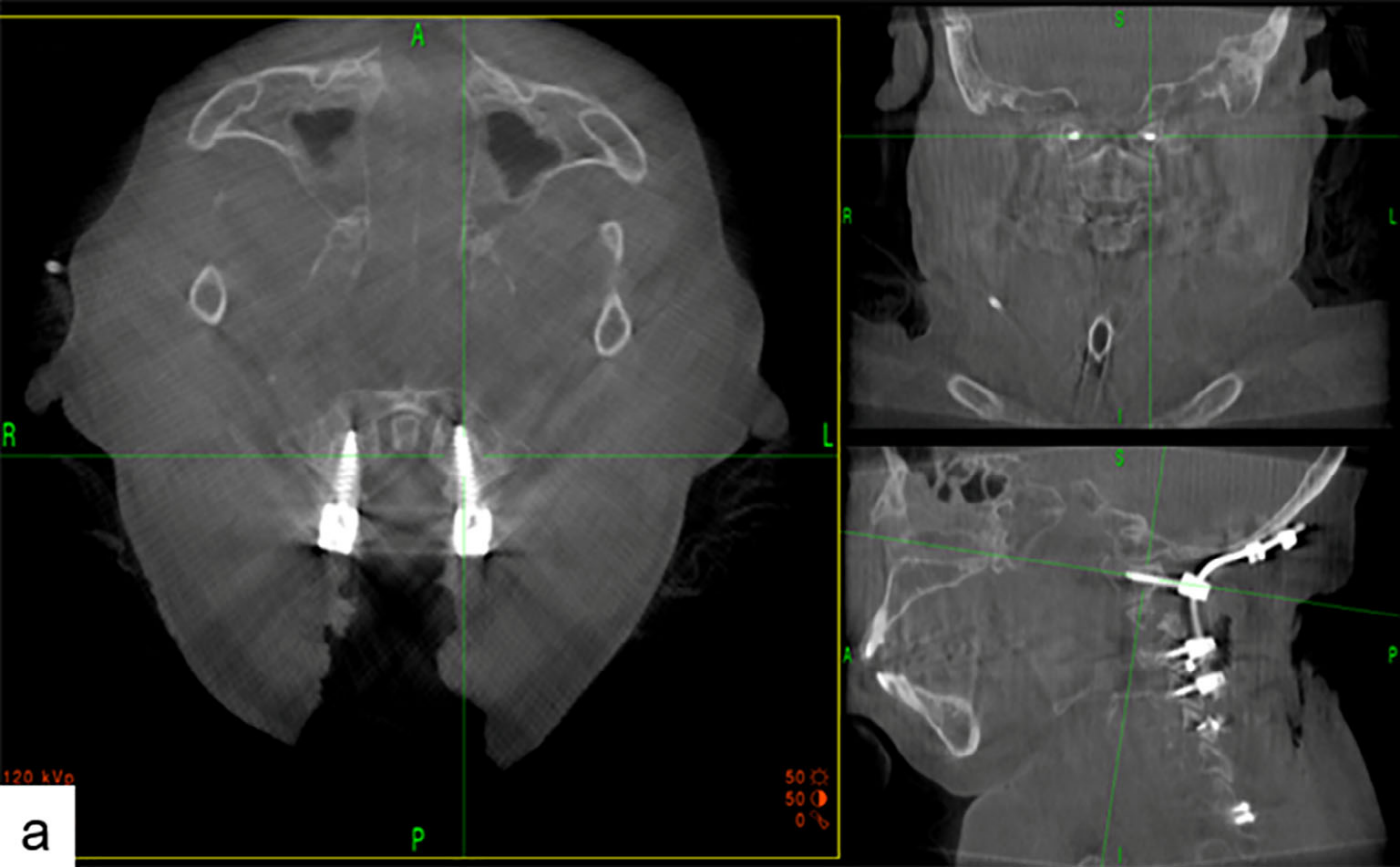
Intraoperative images showed that navigation based on O‐arm device could provide real‐time and precise virtual trajectory, and screws can be placed accurately *via* the O‐arm machine.

**Figure 5:**
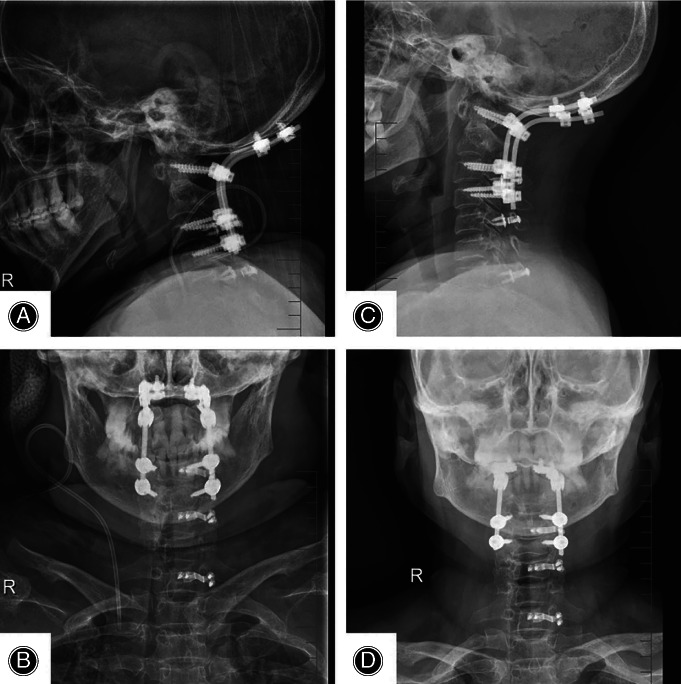
(A, B) Postoperative X‐ray showed accurate position of all the screws and occipitocervical fusion was performed very well. (C, D) X‐ray showed that all screws remained a satisfactory position and no displacement occurred. Occipitocervical fusion had achieved perfect surgical effect.

**Figure 6:**
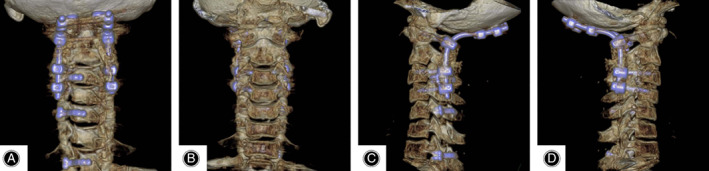
(A, B, C, D) CT reconstruction showed that all the screws penetrated the pedicle into vertebral body accurately from different directions and reconstructed the stability of spinal column. Spinal cord decompression was performed at the compression segment.

**Figure 7:**
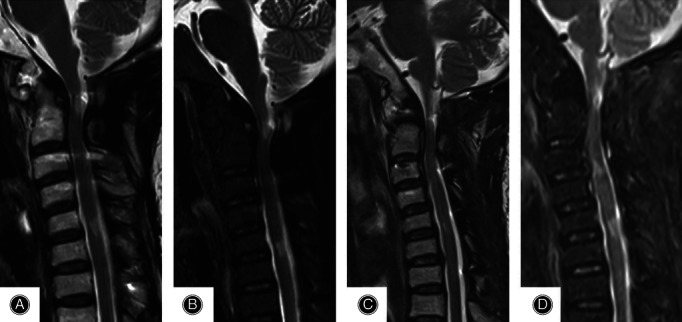
(A, B) MRI showed that the compression of spinal cord was resolved half one year after surgery. (C, D) MRI showed that the spinal cord remained uncompressed and the patient achieved perfect surgical results one year after surgery.

No additional immobilizations were attached postoperatively. The improvement rate was 50% according to JOA scoring system. There were no surgical adverse events and the patient had achieved solid fusion at her final follow‐up. Both intraoperative and postoperative images showed that all screws remained in perfect position (Figs 4, 5, 6, 7).

## Discussion

Occipitocervical fusion may be indicated for diseases that render craniocervical junction unstable. Since rigid fixation replaced nonrigid methods, the procedures of screw placement have continued to be modified for better biomedical stabilities. Among them, the cervical pedicle screw was reported to be able to provide satisfactory biomechanical stability. Claybrooks *et al*. found that C_1_ lateral mass to C_2_ pedicle fixation is superior in lateral bending and axial rotation to C_1_ lateral mass to C_2_ laminar fixation^3^. Furthermore, Ronald *et al*. suggested that cervical pedicle screws could provide the strongest fixation for both initial and salvage applications^4^. Consequently, we employed the cervical pedicle screws for internal fixation in the present study. The individual failure rate of screw/rod techniques reported in the literature was 7.89% and the pseudarthrosis rate and fusion rate were reported to be 2.9% and 93.33%, respectively^10^. In contrast, no screw breakage, displacement, extraction was found till the latest follow‐up in this research, and all patients have already received satisfactory fusions, which demonstrates that fixation through cervical pedicle provides reliable stiffness. Besides, the range of motion of both fixed and adjacent segments in most directions after pedicle fixations was reported to be comparable to that of other procedures^3^, ^11^. Given all this evidence, the cervical pedicle fixation could seemingly be regarded as a favorable internal fixation.

However, due to the complex anatomy of the craniocervical region, cervical pedicle fixation was recognized as one of the most challenged manipulations in orthopaedic surgeries, especially for the atlas. The narrow cervical pedicle crosses between transverse foramen and spinal canal so that any tiny penetration could result in severe complications. Anatomic studies based on radiography or direct measurement reported that the pedicle width and height of cervical vertebrae, especially the upper cervicales, are immensely smaller compared with the lumbar^12^. In terms of angular dimensions of the cervical vertebrae, the end‐plate inclination is angled cephalad at a constant inclination and the pedicle sagittal inclination shows a steep divergence^12^, ^13^. As a result, traditional assessments preoperatively may not meet the demand of precise nailing.

The O‐arm is a novel imaging system that incorporates a flat panel detector and can provide standard fluoroscopic images or three‐dimensional, volumetric CT scans. Compared with traditional fluoroscopy, this technique was considered to have the following advantages: (i) O‐arm provides three‐dimensional reconstructed views of the patient as currently positioned on the operating table and the concern of navigational inaccuracy due to differences in intervertebral alignment between a preoperative image and the intraoperative position is obviated; (ii) the scan was performed only once, which reduces radiation exposure and fluoroscopy time, and as all the staff would leave the operation room in advance, there was nearly no hazard to surgeons; and (iii) O‐arm could interface seamlessly with the Navigation System compared with the three‐dimensional C‐arm fluoroscopy, which could eliminate the time of registration and transferring acquired scans.

With guidance of real‐time navigation, surgeons completed all the fixations via cervical pedicle and obtained a favorable accuracy of screw placement. Patients in the present study were frequently accompanied by severe hyperosteogeny due to degeneration, or instability generated from congenital deformity, which increased the difficulty of placing the cervical pedicle screws. The O‐arm provided us the trajectory of screws by setting parameters including the diameter and length, meanwhile, a virtual screw was computed, displaying on reconstructed images. All the screw placements and revisions were performed under direct orientation without the need to rely on bony landmarks. Former studies reported a quite high breach rate ranging from 16.9% to 23% using freehand technique^14^, ^15^, ^16^, while Singh *et al*. reported a misplacement rate as low as 5% to 6.7% with the help of O‐arm navigation^17^, ^18^, which showed that navigation assistance based on O‐arm can significantly improve the accuracy of cervical pedicle screw positioning. To the best of our knowledge, few clinical studies have been published regarding C_1_ pedicle screw placement in clinical practice^19^, ^20^. For the illustrative patient, preoperative CT reconstruction assessment of C_2_ pedicles revealed that bilateral pedicles were obviously narrow, then two screws were inserted through C_1_ pedicles respectively. In another patient, two C_1_ pedicle screws were inserted for the same consideration. All the four C_1_ pedicle screws were critically positioned within the osseous channels with Grade I. Combined with this experience, we deem that the pedicles of the upper cervical should not always be regarded as the forbidden area. Pedicle fixation of upper cervical spine, including the atlas, is feasible with the assistance of O‐arm navigation and is worth being promoted for better biomechanical performance.

Many factors are related to surgical complications, such as the type of instrumentation, underlying pathology, the clinical experience of operator and postoperative treatment. The overall rate of postoperative adverse events was reported to be 52%, among which neurological deterioration accounted for 41% and wound complications such as infection and dehiscence accounted for 30.9%^10^. Malposition of screw is potentially the most crucial reason for serious complications like artery injury and neurologic deficit. Vertebral artery injury rate in OCF is reported ranging from 2.5% to 8.7% in literature^10^, ^21^. In our series, no neurovascular injuries or any other complications were observed during the follow‐up, indicating this surgical procedure could potentially reduce the risk of postoperative adverse events.

This surgical procedure also has good compatibility with other operations. Two cases successfully underwent hybrid surgeries in this study and internal instrumentations did not interfere with each other. Various means of decompression were employed under the support of solid internal fixation. By resorting to the final scan, surgeons had the opportunity to make assessment and correction in the same operative setting. Besides, in terms of operation time, our result is comparable to other surgical procedures performing OCF^22^, even if there were two complex long‐level surgeries.

There are several limitations in our study. First, sample size is small, which is related to the extremely rare and rigorous conditions. Second, this case series aims to provide primary experience so that no group was set for comparisons. Our results will need to be confirmed in larger series and using study design with higher level evidence.

### *Conclusions*

This retrospective case series demonstrates that O‐arm navigation assisted OCF using cervical pedicle fixation is a feasible and safe procedure with a vast range of indications. The O‐arm navigation greatly improves the accuracy of screw placement and makes it possible to gain better biomechanics in CVJ without sacrificing clinical outcomes. Further study containing larger samples was required to provide higher evidence regarding its clinical practice.

## Compliance with Ethical Standards

Yes.

## Ethical Approval

All procedures performed in studies involving human participants were in accordance with the ethical standards of the institutional and/or national research committee and with the 1964 Helsinki Declaration and its later amendments or comparable ethical standards.
